# A New Method for Measuring Structural Limb-Length Discrepancy: A Quasi-Experimental Study

**DOI:** 10.3390/jcm15114309

**Published:** 2026-06-02

**Authors:** Huthaifa Atallah, Titeana Qufabz, Mahmoud Alfatafta, Amneh Alshawabka, Mohamad Samih Yasin, Omar Q. Samarah, Anthony McGarry, Bálint Molics

**Affiliations:** 1 Prosthetics and Orthotics Department, School of Rehabilitation Sciences, The University of Jordan, Amman 11942, Jordan; titeanaqofabz@hotmail.com (T.Q.); m.alfatafta@ju.edu.jo (M.A.); a.alshawabka@ju.edu.jo (A.A.); 2 Division of Orthopaedics, Department of Special Surgery, School of Medicine, The University of Jordan, Amman 11942, Jordan; myasin@ju.edu.jo (M.S.Y.); o.samarah@ju.edu.jo (O.Q.S.); 3 Biomedical Engineering Department, University of Strathclyde, Glasgow G4 0NW, UK; anthony.mcgarry@strath.ac.uk; 4 Department of Sport Physiotherapy, Faculty of Health Sciences, University of Pécs, 7621 Pécs, Hungary; molics.balint@etk.pte.hu

**Keywords:** limb-length discrepancy, clinical measurement, radiography, malleolus

## Abstract

**Background/Objectives:** Limb-length discrepancy (LLD) is a relatively common condition, with structural LLD characterized by true shortening of a bony structure. Although various clinical and radiological methods are available, none rely exclusively on position of malleoli relative to one another. This study aims to introduce a novel method for measuring structural LLD using malleoli in conjunction with a ruler. A quasi-experimental study. **Methods:** Eighteen participants with structural LLD were recruited from an orthopaedic clinic. Each participant underwent four tests: Paired Medial Malleolus Level Test (PMMLT) (new method), measuring tape method (Anterior Superior Iliac Spine (ASIS) to Medial Malleoli (MM), standing-block test, and full-length standing (long bone) radiography. Validity was assessed against radiography. **Results:** The mean ± SD LLD measured by radiography was 2.99 ± 1.69 cm. Corresponding values were obtained with the PMMLT (2.98 ± 1.69 cm), measuring tape method (2.95 ± 1.66 cm), and the standing-block test (2.92 ± 1.73 cm). No significant differences were found among the methods (χ^2^(3) = 3.43, *p* = 0.330). Correlations with radiography were very strong (PMMLT r = 0.996; measuring tape method r = 0.990; standing-block test r = 0.991; all *p* < 0.001). PMMLT showed negligible bias (−0.01 cm), the narrowest 95% limits of agreement (−0.32 to 0.30 cm), and the lowest RMSE (0.15 cm). **Conclusions:** The PMMLT demonstrates accuracy and practicality while being simple, cost-effective, and free of radiation exposure, making it a valuable alternative for clinical assessment of LLD in settings where imaging is unavailable or contraindicated.

## 1. Introduction

Limb-length discrepancy (LLD) is a relatively common condition, with a prevalence estimated at 40–70% in the general population [1]. Knutson et al. (2005) reported that structural LLD may be present in up to 90% of individuals [2]. In their study, 41.3% of participants demonstrated a discrepancy of 0–4 mm, 37.4% had 5–9 mm, 20% had >9 mm, 15% had 10–14 mm, and 6.4% had >14 mm [2].

LLD can be classified into structural and functional types [1,3]. Structural (anatomical) LLD is characterized by true shortening of a bony structure of the lower limb, which may result from congenital or acquired causes. Congenital causes include hip dislocation or growth deficiencies of the lower extremity, whereas acquired causes may involve hip or knee replacement, trauma, or infection [1,3]. Functional (apparent) LLD occurs when the osseous lengths of the lower limbs are equal, but an apparent difference arises due to altered mechanics, such as pelvic obliquity or hip and knee contractures. The present study focuses on structural LLD.

From a clinical perspective, accurate detection of LLD is essential, as discrepancies greater than 2 cm are associated with altered gait mechanics, compensatory scoliosis, joint pain, and degenerative changes [4,5]. Both clinical and radiological methods are used to measure LLD; however, there is no consensus regarding their validity and reliability [3]. Clinical methods include direct tape measurement [6], the standing-block test [7], Iliac crest palpation with book correction (ICPBC) [8], hand-held pelvic-levelling devices [9,10], prone and supine leg check methods [11], measurement of the distance between the malleoli and the floor [6], and palpation and visual assessment [12]. Radiological methods are radiography with a full-length standing antero-posterior [13] or with pelvic landmarks [14], Computed tomography (CT) [15], ultrasound [16], Magnetic resonance imaging (MRI) [17], picture archiving and communication system (PACS) [18], and bi-planar imaging system EOS [19].

In radiological methods, the length of the entire lower limb from the pelvis and hip to the floor can be captured directly [20]. Alternatively, it can be calculated as the sum of back-foot height, tibial and femoral lengths, and acetabular height [19]. Back-foot height refers to the vertical distance from the plantar surface of the heel to the floor, representing the contribution of the foot and ankle to overall limb length. Clinical methods, in contrast, rely on bony prominences to measure LLD. Commonly used landmarks include the anterior superior iliac spine (ASIS) to the medial malleolus (MM) or lateral malleolus (LM) [6], ASIS to the umbilicus [21], ASIS to the xiphoid process [21], pubis to MM [22], soles of the feet [23], iliac crests height [10], and acetabulum to calcaneus [12]. The malleoli have also been used for visual inspection of LLD [11] or for measuring the distance relative to the floor [6]. However, no method has utilized the malleoli exclusively to measure LLD in relation to each other, without reference to other bony landmarks or the floor.

Recent advances in medical imaging, including low-dose EOS imaging, computed radiography, artificial intelligence (AI)-assisted image analysis, and radiomics-based segmentation techniques, have improved the precision and efficiency of musculoskeletal assessment. AI models such as convolutional neural networks and automated segmentation methods have also demonstrated utility in the evaluation of bone and soft-tissue structures from radiographic imaging. Nevertheless, despite these advances, clinical non-radiological methods remain relevant in outpatient, pediatric, and low-resource settings where rapid, low-cost, and radiation-free assessment is desirable [13,24,25,26].

Therefore, the aim of this study was to introduce a novel method for measuring structural LLD that relies solely on the malleoli and a simple ruler. Unlike radiological techniques, this method requires no specialized equipment and avoids patient exposure to radiation.

## 2. Materials and Methods

### 2.1. Study Design

A quasi-experimental study was conducted to assess the validity of a new method for measuring structural LLD using only the medial malleoli and a millimetre ruler. This method was compared with two commonly used clinical techniques, the measuring tape method and the standing-block test, and all three methods were validated against the radiographic gold standard.

### 2.2. Participants

Following ethical approval from the Jordan University Hospital Institutional Review Board (IRB-JUH) at the University of Jordan (UoJ) (10/2023/3/971), Eighteen participants were recruited from the orthopaedic clinic. Individuals with anatomical (structural) LLD were included, whereas those with functional (apparent) LLD were excluded. All participants provided written informed consent.

### 2.3. Procedure

Limb-length discrepancy (LLD) for each participant was measured by a single observer during a single assessment session, with all clinical measurements performed consecutively using a standardized sequence described below.

**Figure 1:**
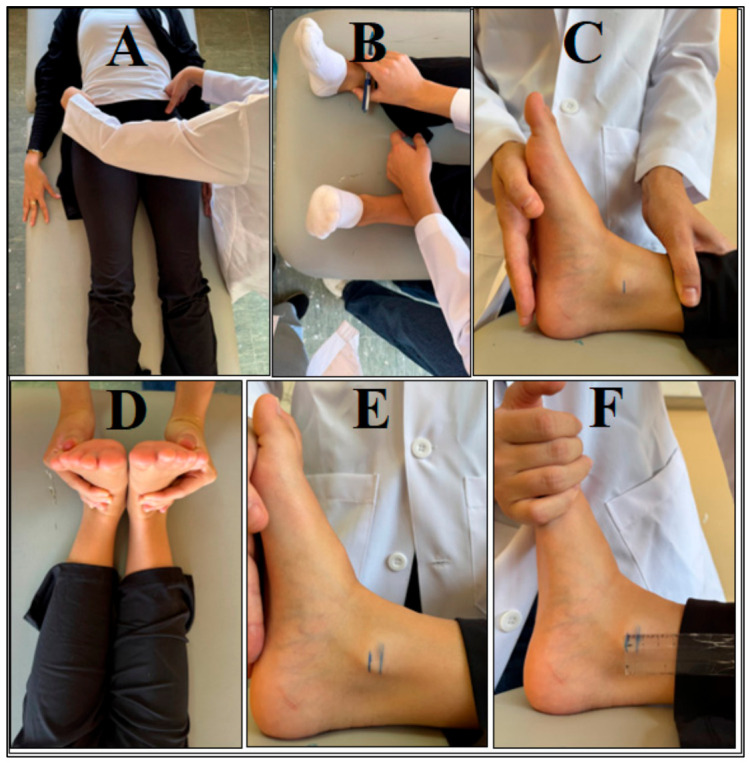
Paired Medial Malleolus Level Test (PMMLT). (**A**) Pelvic levelling, (**B**) Palpation and marking of medial malleoli, (**C**) Marked medial malleoli, (**D**) Feet adducted toward each other to transfer the line, (**E**) Transferred lines, and (**F**) Measurement of the distance. The figure illustrates the complete step-by-step PMMLT procedure to improve methodological clarity and facilitate reproducibility in clinical and research settings.

**Figure 2:**
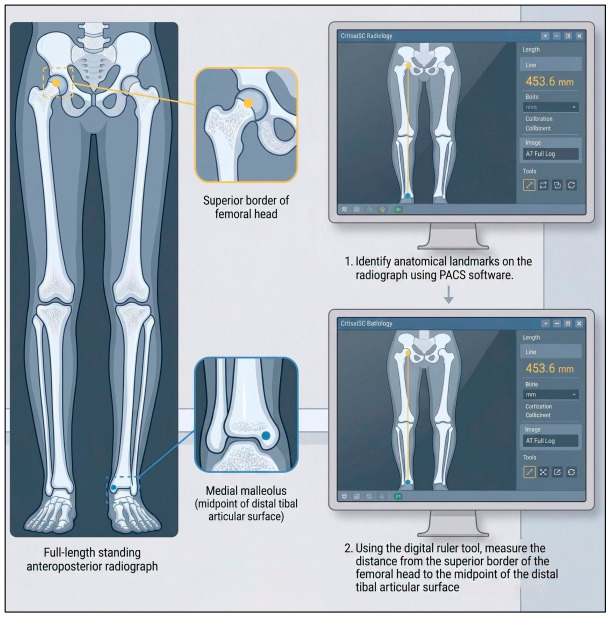
Schematic illustration was included to clarify the anatomical landmarks and measurement procedure used in radiographic assessment.

1. Paired Medial Malleolus Level Test (PMMLT): Participants lay supine with their lower limbs relaxed and aligned in a neutral position. The pelvis was carefully leveled to ensure symmetry of the iliac crests, minimizing measurement error due to pelvic tilt. Pelvic symmetry was confirmed clinically by palpation of bilateral iliac crest height by the same examiner, without the use of an external leveling device. The ankle joints were passively dorsiflexed to the maximum by the examiner (toes pointing toward the ceiling). Participants with severe ankle deformity or fixed contracture limiting positioning were not included in the study. The examiner gently stretched the lower limbs to visualize the peak levels of the medial malleoli. A horizontal line, parallel to the dorsiflexed foot, was drawn on the peaks of the malleoli using a pen. The feet were then adducted toward each other while remaining dorsiflexed. The line from the shorter limb was transferred onto the opposite malleolus, creating two lines on each side. A millimetre ruler was used to measure the distance between the two lines, representing the LLD. Finally, the lines were removed using acetone to avoid examiner bias, as shown in Figure 1.
2. Measuring Tape Method: While the participant remained supine, LLD was measured from the anterior superior iliac spine (ASIS) to the medial malleolus (MM) on each side using a standard measuring tape. The difference between the two measurements was recorded as the LLD value.
3. Standing-Block Test: Custom-designed wooden blocks of varying heights (0.5 cm, 1 cm, 1.5 cm, etc.) were placed under the shorter limb until pelvic levelling was achieved. Iliac crest alignment was palpated to determine equal Limb-length. The total height of the blocks used was recorded as the LLD value.
4. Radiographic Measurement (Gold standard Method): For study purposes, all participants underwent a standardized full-length standing anteroposterior radiograph using a Compax 400 system (General Electric, Milwaukee, WI, USA). LLD was measured from the highest point of the femoral head to the midpoint of the lower articular surface of the ipsilateral tibia using Synapse Radiology PACS software (Version 6.x; Fujifilm Healthcare, Tokyo, Japan). Radiographic measurements were performed using calibrated PACS digital imaging software to reduce magnification-related measurement error. Limb-length discrepancy was measured from the superior aspect of the femoral head to the midpoint of the distal tibial articular surface, as shown in Figure 2. The radiologist performing the measurements was blinded to the clinical results. The anatomical landmarks used for radiographic assessment are illustrated schematically to improve methodological clarity and reproducibility.

### 2.4. Statistical Analysis

Statistical analyses were conducted in IBM SPSS Statistics, Version 22.0 (IBM Corp., Armonk, NY, USA). Normality was assessed with the Shapiro–Wilk test; because distributions deviated from normal, non-parametric methods were applied. To compare limb-length discrepancy (LLD) measurement methods, the Friedman test was used for overall within-subject comparisons across the four techniques: PMMLT, measuring tape method, standing-block test, and radiographic measurement. Kendall’s W was reported as an index of overall rank-order agreement among methods. A Bonferroni adjustment was applied to control for multiple comparisons, with *p* < 0.05 considered statistically significant. For parameters with statistically significant differences, 95% confidence intervals (CIs) for mean differences were calculated. Validity relative to the radiographic reference was examined using Pearson correlation to quantify linear association and Bland–Altman analysis to estimate bias and the 95% limits of agreement (LoA); the root mean squared error (RMSE) was also reported as an index of typical absolute error. An a priori clinical tolerance of ±0.5 cm was defined as the acceptability margin: method–reference discrepancies of 0.5 cm or less were considered clinically negligible, and agreement metrics (bias, 95% LoA, RMSE) were interpreted against this margin. These analyses were selected to demonstrate both the strengths and limitations of the PMMLT relative to radiographic assessment. In addition, the proportion of paired observations within ±0.5 cm of the radiographic was summarized for each method. Proportional bias was evaluated by regressing the method reference difference on the mean of the paired measurements. No priori power analysis was performed, as the study was designed as an exploratory validation study of the PMMLT.

## 3. Results

A total of 18 participants with limb-length discrepancy were included in the study. The majority of participants were male (72.22%), with only five females (27.78%). The mean age of the participants was 10.06 ± 4.28 years, with a range of 4 to 18 years. The mean height was 135.9 ± 24.6 cm, with a range of 90 to 183 cm. The most common cause of LLD was congenital (66.67%), followed by trauma (16.67%), tumor (5.56%), infection (5.56%), and Blount’s disease (5.56%). The right side was more commonly affected (61.11%) than the left (38.89%). In terms of treatment, most participants were not receiving any intervention (55.56%), while 38.89% used an insole and one participant used an ankle foot orthosis (AFO) with height adjustment. None of the participants used an assistive device (Table 1).

Eighteen participants provided complete measurements for all four methods. On the radiographic measurement, mean ± SD LLD was 2.99 ± 1.69 cm. Corresponding values were 2.98 ± 1.69 cm for PMMLT, 2.95 ± 1.66 cm for the measuring tape method, and 2.92 ± 1.73 cm for the standing-block test (Table 2).

The Friedman test showed no statistically significant differences in central tendency across the four methods (χ^2^(3) = 3.43, *p* = 0.330; Kendall’s W = 0.064), indicating limited rank-order differentiation among methods. Given the non-significant overall result, post hoc testing was not pursued, Table 3. Relationships with the radiographic measurement were very strong, with Pearson correlations of r = 0.996 for PMMLT, r = 0.990 for the measuring tape method, and r = 0.991 for the standing-block test (all *p* < 0.001), confirming preservation of patient rank order across methods, Table 3.

**Figure 3:**
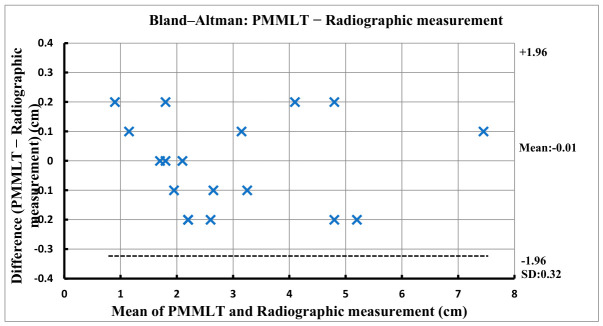
Bland–Altman analysis for PMMLT versus the X-ray showing negligible bias (−0.01 cm), narrow 95% limits of agreement (−0.32 to 0.30 cm), and no evidence of proportional bias (slope −0.002, *p* = 0.936), indicating tight individual-level agreement.

Agreement metrics demonstrated close alignment at the individual level. PMMLT showed negligible bias (mean difference −0.01 cm), 95% limits of agreement −0.32 to 0.30 cm, and RMSE 0.15 cm (1.5 mm); the regression of differences on the mean indicated no proportional bias (slope −0.002, *p* = 0.936), as shown in Figure 3 and Table 4. Measuring tape method also agreed well (bias −0.04 cm; LoA −0.50 to 0.41 cm; RMSE 0.23 cm), as did the standing-block test (bias −0.08 cm; LoA −0.54 to 0.38 cm; RMSE 0.24 cm). Using the pre-specified clinical tolerance of ±0.5 cm as the acceptability margin, the LoA for PMMLT lay entirely within this range. The apparent length and standing block methods were also close to, and largely within, the same range. Moreover, all 18 observations for each method were within ±0.5 cm of the radiographic reference (100%; 95% CI, 82–100). Taken together, these findings indicate clinically acceptable agreement with the reference standard within the predefined tolerance, with PMMLT demonstrating the most favorable error profile and practical interchangeability with the radiographic method for routine LLD assessment.

All three clinic-based methods (techniques) closely mirrored the radiographic measurements, with PMMLT exhibiting the narrowest limits of agreement and the lowest typical error, supporting its use when radiography is unavailable.

## 4. Discussion

The objective of this study was to introduce and validate a novel clinical method, the Paired Medial Malleolus Level Test (PMMLT), for the assessment of structural limb-length discrepancy (LLD) of lower limbs, and to evaluate its validity in comparison with two widely utilized clinical tests, measuring tape method and standing-block test, and the radiographic gold standard (Figure 2). A total of eighteen participants with confirmed LLD were assessed using four different methods, with full-length standing anteroposterior radiography serving as the reference standard (Table 1 and Table 2). For each participant, LLD was measured by a single observer following a consistent sequence of methods. To minimize bias, the PMMLT was conducted first and the radiographic measurement last.

The novelty of the PMMLT lies in its simplified approach, relying exclusively on direct comparison of the paired medial malleoli without dependence on pelvic landmarks or floor reference points. Unlike conventional clinical methods, this technique minimizes examiner-dependent palpation and demonstrated narrower limits of agreement with radiographic measurements.

The measuring tape method, performed from the anterior superior iliac spine (ASIS) to the medial malleolus, yielded a mean LLD of 2.95 ± 1.66 cm (Table 2). It demonstrated a strong correlation with radiography (r = 0.99) (Table 3) but exhibited a higher bias (−0.04 cm) and wider limits of agreement (−0.50 to 0.41 cm) (Table 4). These results are consistent with earlier reports indicating that while the tape method is simple and inexpensive, its accuracy may be compromised by examiner variability, palpation difficulty, and musculoskeletal factors [27,28]. Interestingly, our findings contrast with the study by Sayed-Noor et al., which reported poor correlation (r = 0.21) in patients awaiting total hip arthroplasty [5]. This discrepancy can be explained by the conclusions of the systematic review by Farahmand et al., which noted that the tape method lacks acceptable validity in patients with musculoskeletal disorders [29]. Our exclusion of functional LLD likely contributed to the strong validity observed. However, the predominance of pediatric participants with congenital structural LLD may limit the generalizability of the findings to other clinical populations.

The standing-block test also demonstrated excellent correlation with radiography (r = 0.991) (Table 3), in line with the systematic review by Alfuth et al., which identified the block test as the most useful clinical method for LLD evaluation [3]. However, our analysis revealed that the block test had the greatest bias (−0.08 cm) and widest limits of agreement (−0.54 to 0.38 cm) (Table 4), indicating reduced precision compared to PMMLT. While this method is quick, cost-free, and provides functional information by leveling the pelvis, it remains an indirect assessment and may be influenced by hip or knee contractures. Similarly, PMMLT may also be influenced by soft tissue asymmetry, obesity, joint deformities, or contractures that can alter malleolar positioning and landmark palpation.

The PMMLT demonstrated the highest validity among the clinical methods tested. It showed virtually no bias (−0.01 cm), narrow 95% limits of agreement (−0.32 to 0.30 cm) (Table 4), and an exceptionally strong correlation with radiography (r = 0.996, *p* < 0.001) (Table 3). Bland–Altman analysis confirmed the absence of proportional bias, and no statistically significant differences were found across the four measurement techniques (χ^2^(3) = 3.43, *p* = 0.330) (Figure 3). These findings indicate that PMMLT not only closely mirrors radiographic results but also falls well within the clinically acceptable ±0.5 cm margin of error for LLD assessment [3,30].

The authors acknowledge that radiographic assessment remains the gold standard and provides greater anatomical precision than clinical methods. The strong correlations observed in this study may be related to the standardized protocol, exclusion of functional LLD, and the relatively homogeneous sample. Therefore, the findings should be interpreted as demonstrating strong clinical agreement rather than equivalence to radiographic precision.

Both the measuring tape and standing-block methods remain clinically useful, but our findings highlight their limitations in precision compared with PMMLT. The PMMLT, by relying only on the medial malleoli and a millimeter ruler, reduces subjectivity associated with ASIS palpation and iliac crest leveling. Compared with previously described tape and block methods, PMMLT may simplify landmark identification; however, it still depends on accurate patient positioning and clinical palpation. It is simple, cost-effective, entirely radiation-free, and requires minimal equipment, making it particularly valuable for pediatric populations and in low-resource or outpatient settings. These advantages suggest that PMMLT could serve as a suitable alternative when radiographic evaluation is unavailable or contraindicated. Radiographic imaging remains the gold standard for comprehensive assessment of LLD, particularly in patients with associated angular deformities, where full-length standing radiographs provide detailed anatomical evaluation (Figure 2). Recent developments such as low-dose imaging systems, AI-assisted analysis, and radiomics-based techniques may further improve imaging precision and workflow efficiency. However, these approaches still require specialized equipment, imaging facilities, technical expertise, and greater cost compared with simple clinical assessment methods such as PMMLT. However, despite its practical advantages, PMMLT remains an indirect clinical assessment and is more susceptible to examiner technique and patient positioning than radiographic methods.

Reliable and radiation-free screening methods are especially beneficial for growing children and adolescents, where frequent monitoring may be required [29]. By combining accuracy, accessibility, and safety, PMMLT holds potential as a routine clinical tool for both initial screening and longitudinal follow-up.

## 5. Conclusions

The Paired Medial Malleolus Level Test (PMMLT) demonstrated a high correlation with full-length standing radiographs in the assessment of structural limb-length discrepancy (LLD). It exhibited narrower limits of agreement and lower measurement error compared with the standard measuring tape and standing-block methods, while maintaining excellent correlation with the radiographic gold standard. Given its accuracy, ease of application, cost-effectiveness, and absence of radiation exposure, PMMLT provides highly accurate and reliable LLD measurements that closely approximate radiographic values and outperform other clinical methods in terms of agreement and error margins. Its simplicity, reproducibility, and radiation-free nature make it a potentially valuable option for the clinical evaluation and follow-up of pediatric patients with structural LLD, particularly in pediatric care and resource-limited environments where imaging may be unavailable or undesirable.

## 6. Study Limitations

The present study has several limitations. First, the relatively small sample size, drawn from a single center and with a predominance of congenital LLD, may restrict the generalizability of the findings. Second, functional LLD was excluded, limiting the applicability of the proposed method to structural discrepancies only. In addition, the small sample size and predominantly pediatric congenital LLD population limit the statistical power and broader clinical applicability of the findings. Furthermore, PMMLT does not evaluate associated angular deformities, which may require full-length standing radiographic assessment. Moreover, PMMLT, like other external landmark methods, is an indirect measure of bone length, whereas radiography directly visualizes osseous structures with minimal magnification errors. Despite the strong agreement observed, PMMLT should not be interpreted as having the same absolute precision as radiographic assessment. The accuracy of PMMLT also depends on meticulous patient positioning, including supine alignment with a leveled pelvis and 90° ankle dorsiflexion, as deviations in technique may introduce variability. In addition, pelvic leveling and ankle positioning were performed clinically and may remain susceptible to examiner-dependent variability. Future research should therefore assess the inter- and intra-rater reliability of the PMMLT in larger and more heterogeneous populations and explore its potential utility in the evaluation of functional LLD.

## Figures and Tables

**Table 1:** Descriptive statistics of participants (n = 18).

Characteristic	Description	n (%)
Gender	Male	13 (72.22)
	Female	5 (27.78)
Age (Year)	Mean ± SD	10.06 ± 4.28
	min–max	4–18
Height (m)	Mean ± SD	135.9 ± 24.6 cm
	min–max	90–183 cm
Side of LLD	Right	11 (61.11)
	Left	7 (55.56)
Etiology	Congenital	12 (66.67)
	Trauma	3 (16.67)
	Tumor	1 (5.56)
	Infection	1 (5.56)
	Other (Blount’s)	1 (5.56)
Treatment	None	10 (55.56)
	Insole	7 (38.89)
	Ankle Foot Orthosis (AFO)	1 (5.56)
Assistive device usage	None	18 (100)

**Table 2:** Limb-length Discrepancy (LLD) across four different methods.

Method	LLD Measurements (Mean ± SD) (cm)
Radiographic Measurement	2.99 ± 1.69
PMMLT	2.98 ± 1.69
Measuring Tape Method	2.95 ± 1.66
Standing-Block Test	2.92 ± 1.73

**Table 3:** Correlation with X-ray (SPSS Bivariate Correlations).

Pair (Method vs. Radiographic Measurement)	Pearson r	Sig. (2-Tailed)
PMMLT—Radiographic measurement	0.996	<0.001
Measuring Tape—Radiographic measurement	0.990	<0.001
Standing-Block—Radiographic measurement	0.991	<0.001

**Table 4:** Agreement of clinical LLD methods with the radiographic reference (x-ray): bias, 95% limits of agreement (LoA), and root mean squared error (RMSE).

Method	Bias (cm)	95% LoA Low (cm)	95% LoA High (cm)	RMSE (cm)
PMMLT	−0.01	−0.32	0.30	0.15
Measuring Tape	−0.04	−0.50	0.41	0.23
Standing-Block	−0.08	−0.54	0.38	0.24

## Data Availability

The datasets used and/or analyzed during the current study are available from the corresponding author on reasonable request.

## Abbreviations

The following abbreviations are used in this manuscript:

LLD	Limb-Length Discrepancy
ICPBC	Iliac crest palpation with book correction
CT	Computed tomography
MRI	Magnetic resonance imaging
PACS	picture archiving and communication system
EOS	Bi-planar imaging system
MM	Medial malleolus
LM	Lateral malleolus
UoJ	University of Jordan
PMMLT	Paired Medial Malleolus Level Test
ASIS	Anterior superior iliac spine
Cis	Confidence intervals
SD	Standard deviation
LoA	Limits of agreement
RMSE	Root mean squared error
AFO	Ankle Foot Orthosis
